# Immunomodulatory and Cytotoxic Properties of Enniatin B1 in Porcine Alveolar Macrophages

**DOI:** 10.1002/jat.70114

**Published:** 2026-02-19

**Authors:** Sara Frazzini, Francesca Caloni, Paola Fossati, Giancarlo Ruffo, Luciana Rossi

**Affiliations:** ^1^ Department of Veterinary Medicine and Animal Science (DIVAS) Università degli Studi di Milano Lodi Italy; ^2^ Department of Environmental Science and Policy (ESP) Università degli Studi di Milano Milan Italy

**Keywords:** Enniatin B1, immunomodulatory effects, in vitro, porcine alveolar macrophages, species‐specific model

## Abstract

This study investigates the immunomodulatory effects of Enniatin B1 (ENNB1), an emerging mycotoxin, on porcine alveolar macrophages (PAMs), a species‐specific model relevant to human innate immunity. PAMs were exposed to increasing ENNB1 concentrations (0.5–6 μM) over 6, 24, and 48 h. Results showed a biphasic response: ENNB1 initially enhanced cell viability and induced upregulation of pro‐ and anti‐inflammatory cytokines (e.g., IL‐1β, IL‐6, IL‐10, TNF‐α), COX enzymes, and antioxidant gene GPX‐2. However, prolonged exposure caused downregulation of these markers and reduced cell viability, indicating cytotoxic and immunosuppressive effects. The temporal and concentration‐dependent responses suggest that ENNB1 modulates macrophage function by altering the immune response, with early immune activation followed by immune impairment. These findings provide novel insights into ENNB1’s potential to alter immune homeostasis and highlight the need for further toxicological studies for a correct evaluation of the risk.

## Abstract 2

This study explores the immunomodulatory effects of ENNB1 on PAMs. ENNB1 induced a biphasic, time‐ and dose‐dependent response: initial immune activation with increased cytokine expression and cell viability, followed by cytotoxicity and immune suppression. These findings suggest a modulation of immune response and reveal ENNB1’s potential to disrupt immune homeostasis, underscoring the need for further toxicological evaluation. Workflow and time‐dependent immunological effects following treatment with ENNB1 on porcine alveolar macrophages (PAMs). After the treatment with ENNB1, an early immune activation phase is observed at 24 h, characterized by increased immune markers. This response is followed by a later phase at 48 h marked by cytotoxicity and immunosuppressive effects, indicated by a downregulation of immune activity.

## 1 Introduction

Emerging mycotoxins, such as enniatins (ENNs), represent a class of secondary mold metabolites that are increasingly gaining attention in food and feed safety, since they are not yet routinely monitored, lack regulatory limits, and are being detected more frequently in food, feed, and environmental matrices (Pérez‐Fuentes et al. 2021, 2022, 2024a, 2024c). Unlike well‐characterized mycotoxins such as aflatoxins or ochratoxin A, emerging mycotoxins remain unregulated, as no tolerable daily intake (TDI) or maximum levels have been officially established due to insufficient toxicological and exposure data (Behr et al. 2025). This knowledge gap makes them particularly relevant for risk assessment.

Among the ENNs, Enniatin B1 (ENNB1) has attracted increasing interest in recent years. Although initially less studied than other ENNs (De Felice et al. 2023; Prosperini et al. 2017), ENNB1 is now recognized as one of the most frequently occurring analogues (Fraeyman et al. 2017). It has been reported in a wide variety of commodities (Giannioti et al. 2023; Santini et al. 2012), including cereals, cereal‐derived products, cheese, and even infant food (Pérez‐Fuentes et al. 2024b; Rodríguez‐Cañás et al. 2023). Although current dietary risk assessments indicate negligible risk in some cases (Ji et al. 2024), its wide distribution in widely consumed food categories, including those intended for susceptible populations food categories, highlight its relevance for food safety.

Toxicological data indicate that ENNB1 exerts multiple adverse effects in vitro and in vivo. It has been shown to impair cell viability (Ivanova et al. 2017; Meca et al. 2011; Prosperini et al. 2014), alter cell cycle, induce oxidative stress, disrupt mitochondrial membrane potential, and exert genotoxic and estrogenic effects (Chiminelli et al. 2022). More recent studies revealed that ENNB1 is not a Ca^2+^ ionophore, contrary to previous assumptions (De Felice et al. 2023; Pérez‐Fuentes et al. 2024c), but it may affect cholesterol biosynthesis pathways (Coulet et al. 2024; Sy‐Cordero et al. 2012) and interact with efflux transporters (Dornetshuber et al. 2009; Ivanova et al. 2010). It has also been reported to induce reproductive toxicity (Shen et al. 2024) and cytotoxicity in neuronal cells (Pérez‐Fuentes et al. 2021), as well as negative effects on aquatic organisms (De Felice et al. 2025). Importantly, ENNB1 may also modulate the immune system. While in vivo studies in mice suggest immunomodulatory activity (Behr et al. 2025; Shen et al. 2024), few in vitro data on ENNB1 immunotoxicity are currently available, highlighting an important knowledge gap.

In this context, porcine alveolar macrophages (PAMs) represent a relevant translational model to investigate immunotoxic effects. PAMs play a crucial role in innate immunity, being responsible for pathogen recognition, phagocytosis, and cytokine production (Balmelli et al. 2005). Functionally, they share close similarities with human alveolar macrophages, including cytokine response profiles and receptor expression (Fairbairn et al. 2011; Meurens et al. 2012; Pabst 2020). This makes them a suitable system to evaluate the potential of ENNB1 to alter immune function, especially in relation to respiratory and systemic inflammation.

The present study aimed to evaluate the immunotoxicological effects of ENNB1 in PAMs, focusing on its ability to modulate inflammatory responses in a time‐ and dose‐dependent manner. For this purpose, cells were exposed to ENNB1 concentrations ranging from 0 to 6 μM, a range widely used in in vitro studies to capture both early modulatory and overt cytotoxic effects (De Felice et al. 2023; Fraeyman et al. 2017; Prosperini et al. 2017). Although these concentrations exceed typical human exposure levels, which is usually in the ng/g range in food and biological samples (Castell et al. 2025), higher doses are often necessary in vitro to elicit measurable responses in the absence of absorption, distribution, metabolism, and excretion (ADME) processes. Thus, the applied concentrations should be regarded as mechanistic and exploratory, designed to characterize cellular pathways rather than to directly simulate real‐life exposure scenarios.

By integrating this approach, the study aims to provide new insights into the immunotoxic profile of ENNB1 and contribute to the broader understanding of emerging mycotoxins as contaminants of concern for food safety and public health.

## 2 Materials and Methods

### 2.1 Collection of PAMs

PAMs were obtained from clinically healthy piglets intended for the commercial meat industry, in accordance with Directive 2010/63/EU on the protection of animals used for scientific purposes (European Commission 2010). Prior to cell collection, the animals underwent routine ante mortem and postmortem examinations by official veterinary staff to rule out the presence of clinical signs or infectious diseases.

To isolate PAMs, the bronchoalveolar lavage technique was used according to Frazzini et al. (2025). In brief, following humane euthanasia, lungs with the trachea intact were excised, and 100 mL of ice‐cold phosphate‐buffered saline (PBS) without calcium and magnesium was gently flushed through the trachea. After careful manual massage of the lung tissue, the resulting lavage fluid was collected through sterile gauze into 50 mL centrifuge tubes, and cells were sedimented by centrifugation at 400 × g for 10 min at 4°C.

The resulting cell pellet was treated with ACK lysing buffer (Thermo Fisher, MA, USA) to eliminate erythrocytes and subsequently resuspended in RPMI‐1640 culture medium, enriched with 1% antibiotics (100 mg/mL penicillin and 100 mg/mL streptomycin; Mediatech, Manassas, VA, USA) and 5% fetal bovine serum (HyClone Laboratories, Logan, UT, USA). Cell viability was assessed by trypan blue exclusion assay (Sigma‐Aldrich, St. Louis, MO, USA), and viable cells were quantified using a hemocytometer (Fisher Scientific, Pittsburgh, PA, USA).

### 2.2 Cell Culture and Experimental Design

Freshly isolated PAM cells were seeded at 10^6^ cells/mL in 24‐well cluster plates and incubated for 1 h at 37°C with 5% CO_2_ to allow macrophages to adhere to the well surface. The nonadherent cells were washed away, and the adherent macrophages were treated with fresh, prewarmed RPMI‐1640 medium containing different concentrations of ENNB1 (Sigma‐Aldrich, St. Louis, MO, USA). The toxin was tested following a factorial arrangement in a randomized complete block design, including three time points (6, 12, and 24 h) × 5 ENNB1 concentrations (0, 0.5, 1.5, 3, 6 μM). All conditions were tested in both technical replicates (multiple measurements of the same biological sample) and biological replicates (independent samples derived from different biological sources) to ensure the reproducibility and reliability of the result.

### 2.3 Cristal Violet Assay

After each incubation time point, cell adherence was evaluated using a crystal violet assay, based on the method described by Khatua et al. (2022). Briefly, cells were rinsed with PBS and incubated at 37°C for 30 min under agitation with a 0.2% (w/v) crystal violet solution containing 2% (v/v) ethanol in PBS. After incubation, cells were washed again with PBS and allowed to dry completely. The retained dye was then solubilized by adding 200 μL of 33% (v/v) acetic acid in distilled water.

Absorbance was measured at 540 nm using a microplate reader (BioTek Epoch, Agilent, Santa Clara, CA, USA). As an indirect indicator of cell viability, the number of adherent cells was assessed by crystal violet staining and expressed as a percentage relative to untreated controls, calculated using the following formula:

$$\left(\frac{\mathrm{OD}\;\text{sample}}{\mathrm{OD}\;\text{control}}\right)\times 100$$

### 2.4 MTT Assay

Viability was assessed using the MTT assay (Promega Corporation, Madison, WI, USA) according to the manufacturer's instructions. Briefly, after each time point the cells were washed with PBS and 100 μL of fresh medium was added. Subsequently, 15 μL of Dye Solution was added to each well and the plates were incubated at 37°C with 5% CO_2_ for 4 h. After that, 100 μL of Stop solution was added to each well, and the plates were incubated for 1 h. Finally, absorbance was recorded at 570 nm using a microplate reader (BioTek Epoch, Agilent, Santa Clara, CA, USA). The negative control wells containing growth media without cells were used as blanks and subtracted from the absorbance for each sample. The OD of positive control wells containing cells in growth media without treatment was used as a standard and set as 100% viability. The relative viability was calculated as follows:

$$\left(\frac{{\mathrm{OD}}_{\text{sample}}-{\mathrm{OD}}_{\text{blank}}}{{\mathrm{OD}}_{\text{control}}}\right)\times 100$$

### 2.5 RNA Extraction, Retro‐Transcription, and Quantitative Real‐Time PCR

Gene expression was evaluated after 24 and 48 h of incubation, as transcriptional changes were expected to require longer exposure times to become consistently detectable. At these time points, cells were detached with Trypsin–EDTA Solution (Sigma‐Aldrich Co., St. Louis, MO, USA), collected, and stored at −80°C for RNA extraction. Total RNA was extracted from PAMs using the RNeasy Mini Kit (Qiagen, Hilden, Germany) according to the manufacturer's guidelines. RNA concentration and purity were assessed by measuring absorbance at 230, 260, and 280 nm with a NanoDrop 2000 spectrophotometer (Thermo Fisher Scientific, Massachusetts, USA). RNA samples were stored at −80°C until use.

For cDNA synthesis, 1 μg of total RNA from each sample was reverse transcribed using the iScript cDNA Synthesis Kit (Bio‐Rad, Richmond, CA, USA), following the manufacturer's instructions. The reverse transcription was performed in a total reaction volume of 20 μL under the following thermal conditions: 5 min at 25°C, 20 min at 46°C, and 1 min at 95°C.

Quantitative PCR was performed using a CFX Opus 96 real‐time PCR system (Bio‐Rad, Richmond, CA, USA). Each 20 μL reaction mixture included 10 μL of 2X SsoAdvanced Universal SYBR Green Supermix (Bio‐Rad, Richmond, CA, USA), 500 nM of each primer (Table 1), and 10 ng of cDNA. The cycling program was as follows: initial denaturation at 95°C for 30 s, followed by 40 amplification cycles consisting of 15 s at 95°C and 30 s at 60°C. Reactions were considered negative when the cycle threshold (Ct) value exceeded 37.

**TABLE 1:** Oligonucleotide sequences used for the qRT‐PCR assay.

Gene	Nucleotide sequence	Accession number	References
IL‐1β	Fw: TGCCAACGTGCAGTCTATGG	NM_ 214055	Smolinska et al. (2019)
	Rv: TGGGCCAGCCAGCACTAG		
IL‐6	Fw: TGGGTTCAATCAGGAGACCT	AM501528.1	Daniłowicz et al. (2008)
	Rv: CAGCCTCGACATTTCCCTTA		
IL‐8	Fw: TGCCTTCTTGGCAGTTTTCCT	NM_213867	Zeitz et al. (2017)
	Rv: TGGGGTCCACTCTCAATCACT		
IL‐10	Fw: TGAAGAGTGCCTTTAGCAAGCTC	NM_214041.1	Zeitz et al. (2017)
	Rv: CTCATCTTCATCGTCATGTAGGC		
IL‐17A	Fw: CTCCAAACGCTTCACCTCAC	NM_001005729.1	Ryan et al. (2012)
	Rv: GCATTGATACAGCCCGAG		
TNF‐α	Fw: CCACGCTCTTCTGCCTACTGC	NM_ 214022	Chen et al. (2019)
	Rv: CGACGGGCTTATCTGAGGTTTG		
TGF‐β	Fw: TGATGTCACCGGAGTTGTGC	NM_214015.2	Dell'Anno et al. (2024)
	Rv: GGCCAGAATTGAACCCGTTA		
COX‐1	Fw: GGAGCGGGTACTGGATGAAC	EF568726	Caprarulo et al. (2023)
	Rv: CACCTGCAAGGGTGTAGGGAG		
COX‐2	Fw: AAGACGCCACTTCACCCATC	AF304201	Kijas and Andersson (2001)
	Rv: TCCATTGTGCTAGTGTGTGTCA		
GPX‐2	Fw: GGAGATCCTGAACAGCCTCA	DQ898282	Caprarulo et al. (2023)
	Rv: GCGAAGACAGGATGCTCATT		
STAT3	Fw: GTGATGCTTCCCTGATTGTG	NM_001044580.1	Yang et al. (2017)
	Rv: GCAAGGAGTGGGTCTCTAGG		
RELA p65	Fw: CATGAGCTCGTGGGGAAAGA	AK350093.1	Li et al. (2018)
	Rv: CACACTGGATCCCCAGGTTC		
IκB	Fw: TGCAGGCCACCAACTACAAT	Z35483.1	Li et al. (2018)
	Rv: TCAACAAGAGCGACACCAGG		
NFKBIA	Fw: TGGTGTCGCTCTTGTTGAAGTGTG	NM_001005150.1	de Martin et al. (1995)
	Rv: GCTGCTGTATCCGAGTGCTTGG		
GAPDH	Fw: ACCCAGAAGACTGTGGATGG	NM_001206359.1	Luo et al. (2018)
	Rv: AAGCAGGGATGATGTTCTGG		

Abbreviations: COX1, cytochrome c oxidase subunit I; COX2, cytochrome c oxidase subunit II; GAPDH, glyceraldehyde‐3‐phosphate dehydrogenase; GPX‐2, glutathione peroxidase 2; IκB, inhibitor of kappa B; IL‐1β, interleukin‐1 β; IL‐6, interleukin‐6; IL‐8, interleukin‐8; IL‐10, interleukin‐10; IL‐17A, interleukin‐17A; NFKBIA, nuclear factor of kappa light polypeptide gene enhancer in B‐cells inhibitor, alpha; RELA p65, RELA proto‐oncogene, NF‐kB subunit; STAT3, signal transducer and activator of transcription 3; TGF‐β, transforming growth factor β; TNF‐α, tumor necrosis factor‐α.

Gene expression analysis used a control sample (untreated) as the reference. Relative quantification was performed using the 2^−ΔΔCt^ method, with GAPDH serving as the endogenous reference gene (Oh et al. 2023). Data were normalized and analyzed using CFX Maestro software v2.2 (Bio‐Rad, Richmond, CA, USA). Gene expression analyses were conducted in technical replicates (three independent qPCR measurements of the same sample) and biological replicates (independent cell cultures subjected to the same treatment).

### 2.6 Statistical Analysis

All the data were analyzed using GraphPad Prism, Version 9.0.0 (Boston, MA, USA). Before performing the two‐way analysis of variance (two‐way ANOVA), the normality of the data distribution and residuals was assessed using the D'Agostino–Pearson test. The data were then analyzed to evaluate the effects of treatment, time, and their interaction. Post hoc pairwise comparisons were performed using Bonferroni Sidak's test. The data were reported as the mean ± standard deviation, and differences were considered to be statistically significant at *p* ≤ 0.05.

## 3 Results

### 3.1 Assessment of Adherent Cell Density

**FIGURE 1:**
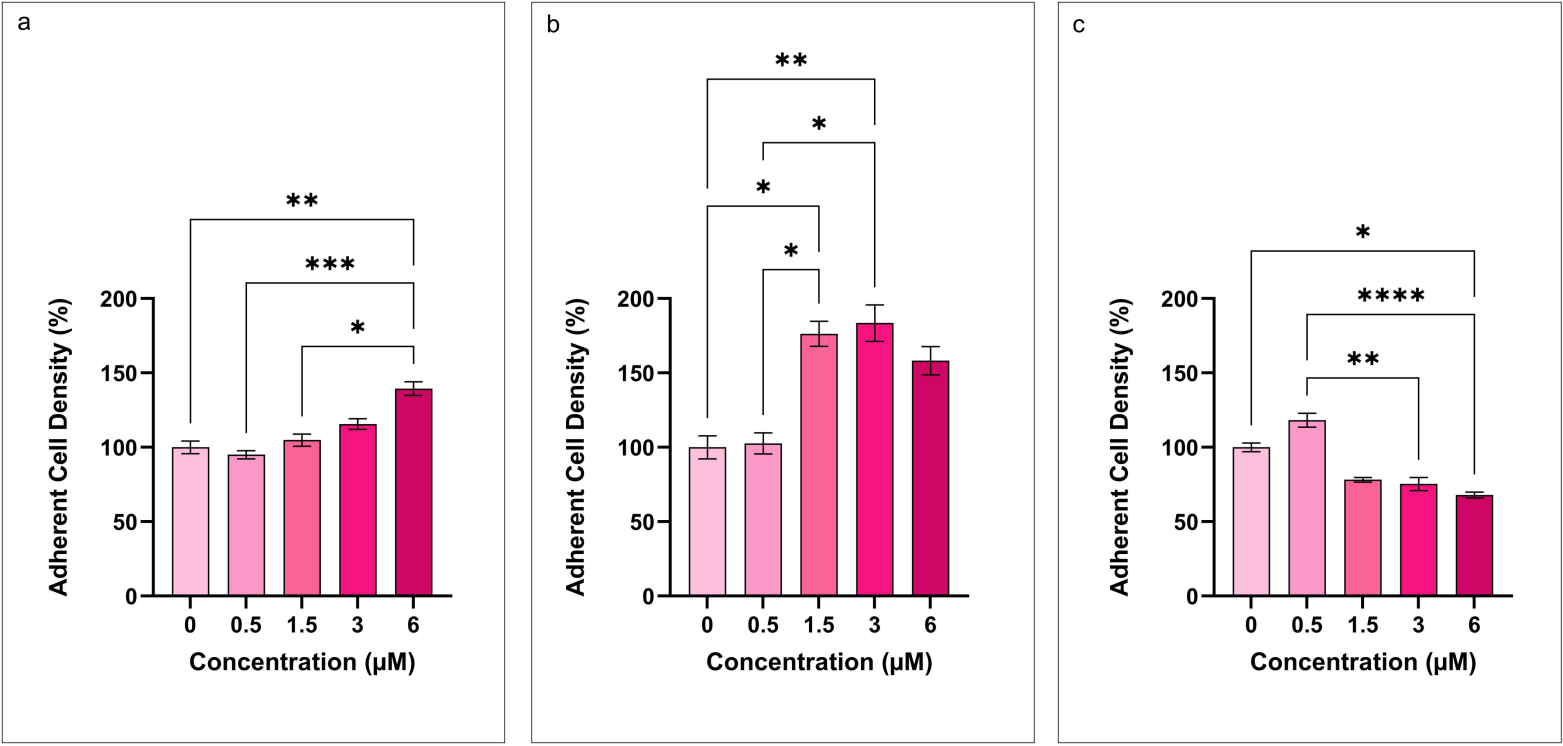
Effect of Enniatin B1 (ENNB1) on the density of adherent porcine alveolar macrophages (PAMs) after (a) 6 h, (b) 24 h, and (c) 48 h of exposure at various concentrations. Cell retention was assessed by crystal violet staining and expressed as a percentage relative to the untreated control (0 μM). Data are presented as percentage viability relative to the untreated control (0 μM). Statistical significance is indicated by asterisks. *p ≤ 0.048; **p ≤ 0.0069; ***p ≤ 0.0001.

After 6 h of incubation (Figure 1a), a moderate but significant increase in cell adherence was observed in a dose‐dependent manner. In particular, when macrophages were co‐cultured with the highest concentration of ENNB1 (6 μM), an increase in adherence of 39.5% was observed with respect to the cells that were not exposed to ENNB1. No significant differences were detected at 3 μM compared with the other tested concentrations, while exposure to 6 μM ENNB1 resulted in significantly higher adherence than the cells co‐cultured with 0.5 and 1.5 μM.

After 24 h of incubation (Figure 1b), the increase in the number of adherent cells became more evident. All concentrations tested (0.5–6 μM) led to a statistically significant enhancement in cell retention compared to the untreated group, with the most notable effects observed at 1.5 and 3 μM, corresponding to increases of 76.3% (*p* = 0.0144) and 83.5% (*p* = 0.0056), respectively. Interestingly, although the signal at 6 μM remained higher than the control, a slight reduction, equal to 13.8%, was noted compared to the peak response at 3 μM.

In contrast, a markedly different pattern was observed at 48 h (Figure 1c), with a concentration‐dependent reduction in the number of adherent cells. While treatment with 0.5 μM ENNB1 resulted in a slight, nonsignificant increase compared to the control, higher concentrations (1.5–6 μM) led to significant decreases in cell retention, indicative of cytotoxic effects. The strongest reduction was recorded at 6 μM, where the number of adherent cells dropped substantially (*p* = 0.0131).

To support the spectrophotometric data, representative phase‐contrast images were acquired under a microscope (Leica DM IL LED, Leica Microsystems Srl, Milan, Italy). These images, presented in Figure S1, are shown for illustrative purposes only and were not used for cell quantification, as spectrophotometric measurement was performed on whole‐well staining.

We acknowledge that under certain treatment conditions, cells exhibited heterogeneous distribution or clustering, which may affect visual interpretation. However, the spectrophotometric analysis remains reliable and reproducible as it integrates the total retained dye signal across the entire well surface, regardless of localized variations in cell distribution.

### 3.2 Cell Viability

**FIGURE 2:**
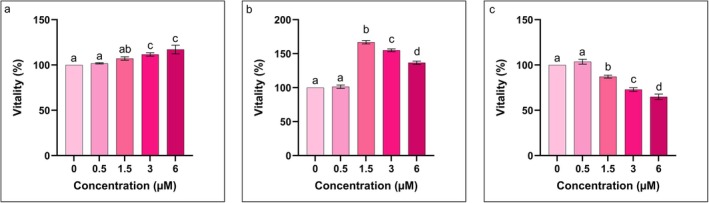
Effect of Enniatin B1 (ENNB1) on the viability of porcine alveolar macrophages (PAMs) after (a) 6 h, (b) 24 h, and (c) 48 h of exposure at various concentrations. Data are presented as percentage viability relative to the untreated control (0 μM). ^a, b, c, d^Different subscripts indicate statistical significance differences.

The effects of ENNB1 on PAM viability were time‐ and dose‐dependent, as shown in Figure 2. After 6 h of incubation (Figure 2a), cell viability remained unchanged at 0.5 μM, whereas a slight but significant increase was detected at 1.5 μM compared to the control (*p* = 0.0331). Higher concentrations (3 and 6 μM) induced a further rise in metabolic activity (*p* = 0.0013 and *p* < 0.0001, respectively).

After 24 h of incubation, ENNB1 induced a clear and concentration‐dependent increase in PAM metabolic activity (Figure 2b). Viability remained comparable to the control at 0.5 μM, whereas exposure to 1.5 μM resulted in a marked and statistically significant elevation (*p* < 0.0001). At higher concentrations, metabolic activity remained significantly elevated versus the control, with 3 μM slightly lower than 1.5 μM (*p* = 0.0003) and 6 μM lower than 3 μM (*p* < 0.0001), indicating a decrease from the maximal response rather than a reduction below baseline. Following 48 h of exposure (Figure 2c), ENNB1 toxicity further intensified. While 0.5 μM did not differ from control, 1.5 μM already induced a clear reduction in viability (*p* = 0.0002), and even more evident decreases were observed at 3 and 6 μM (*p* < 0.0001).

Overall, ENNB1 induced divergent early responses, slight metabolic stimulation at 6 h, followed by a robust, concentration‐dependent reduction in PAM viability at 24 h and a stronger reduction at 48 h, as assessed at the time points.

### 3.3 Gene Expression

**FIGURE 3:**
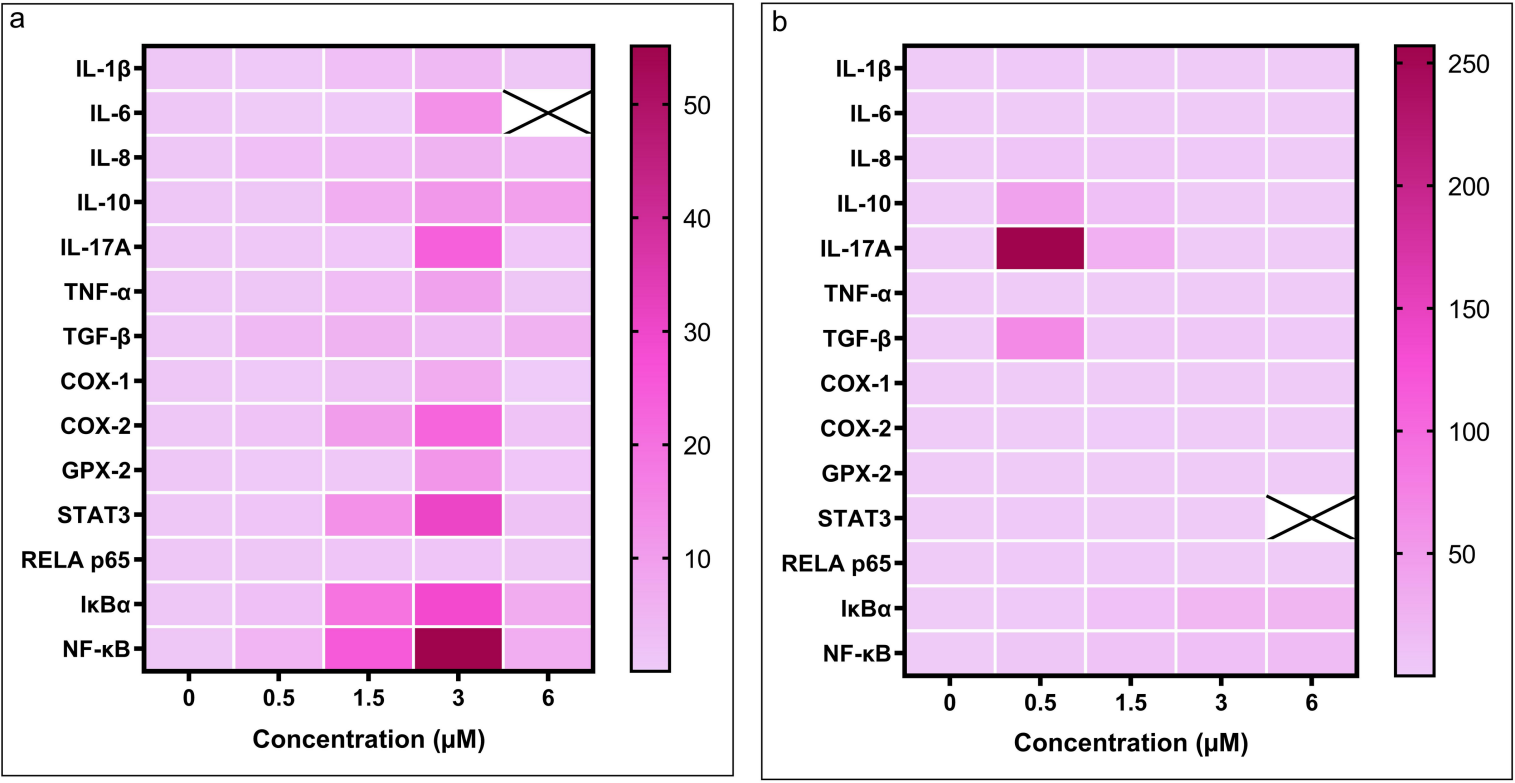
Relative gene expression levels in porcine alveolar macrophages (PAMs) exposed to Enniatin B1 ENNB1 for 24 and 48 h. (a) After 24 h of treatment. (b) After 48 h of treatment. Data are expressed as fold change.

The effects of ENNB1 on gene expression in PAMs after 24 and 48 h of exposure are summarized in Table 2 and illustrated in Figure 3. Overall, a general upregulation of immune‐related and stress‐response genes was observed after 24 h, particularly at 3 μM, while at 48 h, most genes exhibited downregulation or loss of expression, reflecting a time‐dependent biphasic response.

**TABLE 2:** Fold change values for gene expression in response to different concentrations of ENNB1. Data are expressed as changes relative to the untreated control sample (0 μM), which served as the reference for statistical comparison. Data are expressed as mean ± standard deviation.

Genes	0.5 μM	1.5 μM	3 μM	6 μM
	24 h of incubation			
IL‐1β	0.64 ± 0.084^a^	2.76 ± 0.141^b^	4.11 ± 0.071^b^	0.95 ± 0.042^a^
IL‐6	0.05 ± 0.081^b^	0.25 ± 0.154^b^	12.99 ± 0.070^c^	n.d.
IL‐8	2.73 ± 0.028^b^	3.17 ± 0.022^b^	5.32 ± 0.021^c^	3.61 ± 0.014^b^
IL‐10	1.11 ± 0.021^a^	6.52 ± 0.028^b^	11.19 ± 0.007^b^	9.82 ± 0.035^b^
IL‐17A	0.76 ± 0.013^a^	1.15 ± 0.007^a^	23.34 ± 0.008^b^	1.13 ± 0.020^a^
TNF‐α	1.03 ± 0.010^a^	3.24 ± 0.014^b^	9.00 ± 0.007^c^	1.09 ± 0.008^a^
TGF‐β	4.03 ± 0.025^b^	5.38 ± 0.014^b^	3.51 ± 0.010^b^	5.67 ± 0.015^b^
COX‐1	0.65 ± 0.042^a^	2.12 ± 0.021^b^	7.03 ± 0.014^c^	0.02 ± 0.001^d^
COX‐2	1.85 ± 0.007^a^	10.19 ± 0.007^b^	22.38 ± 0.012^c^	1.77 ± 0.005^a^
GPX‐2	0.46 ± 0.021^a^	0.78 ± 0.015^a^	11.82 ± 0.030^b^	1.27 ± 0.021^a^
STAT3	1.74 ± 0.219^a^	13.13 ± 0.020^b^	30.70 ± 0.010^c^	2.09 ± 0.061^a^
RELA p65	1.06 ± 0.570^a^	1.72 ± 0.237^b^	1.52 ± 0.020^c^	1.08 ± 0.453^a^
IκB	2.69 ± 0.359^a^	19.29 ± 0.010^b^	29.14 ± 0.180^c^	7.19 ± 0.049^d^
NFKBIA	5.92 ± 0.010^a^	24.00 ± 0.100^b^	55.14 ± 0.420^c^	6.54 ± 0.59^a^
	48 h of incubation			
IL‐1β	1.77 ± 0.007^a^	0.24 ± 0.014^b^	0.16 ± 0.028^b^	0.04 ± 0.007^b^
IL‐6	0.34 ± 0.014^b^	0.17 ± 0.021^b^	0.15 ± 0.007^b^	0.06 ± 0.007^b^
IL‐8	7.27 ± 0.021^b^	3.08 ± 0.022^c^	2.09 ± 0.008^c^	0.52 ± 0.035^a^
IL‐10	42.35 ± 0.445^b^	12.06 ± 0.035^c^	0.82 ± 0.028^a^	0.26 ± 0.006^a^
IL‐17A	256.91 ± 1.230^b^	28.36 ± 0.679^c^	0.57 ± 0.027^a^	0.045 ± 0.035^a^
TNF‐α	0.56 ± 0.042^a^	0.435 ± 0.049^a^	0.16 ± 0.049^a^	0.16 ± 0.035^a^
TGF‐β	66.85 ± 1.131^b^	3.48 ± 0.572^a^	3.39 ± 0.233^a^	1.55 ± 0.120^a^
COX‐1	0.05 ± 0.071^b^	0.11 ± 0.014^b^	0.08 ± 0.007^b^	0.06 ± 0.014^b^
COX‐2	0.04 ± 0.063^b^	0.01 ± 0.015^b^	0.064 ± 0.176^a^	0.03 ± 0.021^b^
GPX‐2	0.03 ± 0.015^b^	0.11 ± 0.008^b^	0.03 ± 0.008^b^	0.12 ± 0.022^b^
STAT3	1.22 ± 0.501^a^	0.84 ± 0.039^a^	0.55 ± 0.051^b^	n.d.
RELA p65	1.08 ± 0.050^a^	1.11 ± 0.010^a^	0.81 ± 0.035^b^	0.15 ± 0.420^c^
IκB	2.09 ± 0.221^a^	10.67 ± 0.115^b^	20.68 ± 0.060^c^	22.47 ± 0.550^d^
NFKBIA	4.16 ± 0.405^a^	9.19 ± 0.624^d^	12.00 ± 0.010^c^	16.00 ± 0.070^d^

*Note*: ^a,b,c,d^ Different superscript letters within the same row indicate significant differences among concentrations at each time point (p 〈 0.05).

Abbreviations: COX1, cytochrome c oxidase subunit I; COX2, cytochrome c oxidase subunit II; GPX‐2, glutathione peroxidase 2; IκB, inhibitor of kappa B; IL‐1β, interleukin‐1 β; IL‐6, interleukin‐6; IL‐8, interleukin‐8; IL‐10, interleukin‐10; IL‐17A, interleukin‐17A; NFKBIA, nuclear factor of kappa light polypeptide gene enhancer in B‐cells inhibitor, alpha; RELA p65, RELA proto‐oncogene, NF‐kB subunit; STAT3, signal transducer and activator of transcription 3; TGF‐β, transforming growth factor β; TNF‐α, tumor necrosis factor‐α.

After 24 h, the majority of analyzed genes were significantly upregulated compared to the control, with peak responses generally detected at 3 μM ENNB1 (Figure 3a). IL‐1β was significantly increased at 1.5 μM (*p* < 0.0001) and 3 μM (*p* < 0.0001), while expression at 0.5 μM (*p* = 0.0641) and 6 μM (*p* = 0.8856) remained similar to control. IL‐6 was strongly induced at 3 μM (*p* < 0.0001) but markedly reduced at 6 μM, with expression levels falling below the threshold of reliable detection. IL‐8 showed a significant and dose‐dependent upregulation at 0.5, 1.5, and 3 μM (*p* < 0.0001), remaining above control levels even when slightly reduced at 6 μM (*p* < 0.0001). Among anti‐inflammatory cytokines, IL‐10 was significantly upregulated at 1.5, 3, and 6 μM (all *p* < 0.0001). IL‐17A was markedly induced only at 3 μM (*p* < 0.0001), while TNF‐α showed significant dose‐dependent upregulation at 1.5 μM (*p* < 0.0001) and 3 μM (*p* < 0.0001), returning to control‐like levels at 6 μM (*p* = 0.0518). TGF‐β was significantly increased at all tested concentrations (*p* ≤ 0.0008). Consistent with cytokine activation, genes involved in inflammatory signaling were also significantly modulated at 24 h. STAT3 expression was significantly increased at 1.5 and 3 μM (*p* < 0.0001), reaching a pronounced peak at 3 μM, whereas expression at 6 μM was significantly reduced compared to intermediate doses (*p* < 0.0001). RELA p65 was significantly upregulated at 1.5 and 3 μM (*p* < 0.05), indicating activation of NF‐κB–dependent transcription. Accordingly, NF‐κB expression was strongly induced at 1.5 and 3 μM (*p* < 0.0001), while a significant reduction was observed at 6 μM (*p* < 0.01). In parallel, IκBα expression was markedly increased at 1.5 and 3 μM (*p* < 0.0001), suggesting activation of the canonical NF‐κB pathway together with induction of its negative feedback regulator. Regarding enzymatic markers, COX‐1 was significantly upregulated at 1.5 μM (*p* < 0.0001) and 3 μM (*p* < 0.0001), while expression was absent at 6 μM. COX‐2 displayed progressive upregulation up to 3 μM (*p* < 0.0001), but expression decreased significantly at 6 μM (*p* < 0.0001). GPX‐2 was significantly induced only at 3 μM (*p* < 0.0001), while at 0.5, 1.5, and 6 μM, expression did not differ from control (*p* > 0.05).

At 48 h, a general shift towards downregulation was observed (Figure 3b). IL‐1β showed a nonsignificant increase at 0.5 μM (*p* = 0.0923) but was significantly downregulated at higher concentrations (*p* < 0.05). IL‐6 exhibited dose‐dependent suppression across all concentrations. IL‐8 was significantly upregulated at 0.5 μM (*p* < 0.0001), but expression decreased progressively at 1.5 and 3 μM (*p* < 0.0001) and was slightly below control at 6 μM (*p* = 0.9941). For anti‐inflammatory mediators, IL‐10 showed strong induction at 0.5 μM (*p* < 0.0001), followed by significant downregulation at 1.5 μM (*p* < 0.0001). At 3 and 6 μM, expression returned to control‐like levels. IL‐17A was highly upregulated at 0.5 and 1.5 μM (*p* < 0.0001), while it was comparable to control at 3 μM and almost absent at 6 μM. TNF‐α exhibited a progressive, nonsignificant reduction as ENNB1 concentration increased (*p* > 0.05). TGF‐β was strongly induced at 0.5 μM (*p* < 0.0001), but expression decreased dose‐dependently and returned to control levels at higher concentrations (*p* > 0.05). In line with these trends, STAT3 expression at 48 h was significantly reduced compared to 24 h and remained close to baseline across all concentrations (*p* < 0.05). RELA p65 and NF‐κB also exhibited significant downregulation at 48 h, particularly at the highest concentration, where expression fell below control values (*p* < 0.01). Conversely, IκBα expression remained significantly elevated at 1.5, 3, and 6 μM (*p* < 0.0001), indicating sustained inhibitory control of NF‐κB signaling and contributing to the overall suppression of inflammatory gene expression observed at this later time point. Finally, COX‐1 expression was absent at 0.5 μM and significantly downregulated at 1.5, 3, and 6 μM (all *p* < 0.0001). COX‐2 was negligible at 0.5, 1.5, and 6 μM (*p* < 0.0001), while expression at 3 μM was comparable to control (*p* = 0.0624). GPX‐2 was undetectable at 0.5 and 3 μM, while residual expression was observed at 1.5 and 6 μM, though still significantly lower than the control (*p* < 0.0001).

## 4 Discussion

This study was designed to investigate the immunomodulatory action of ENNB1 on PAMs, considering both time‐ and dose‐dependent effects on cellular adherence, viability, and gene expression. Our findings indicate that ENNB1 can exert both cytoprotective and cytotoxic effects, as well as significant modulation of immune‐related gene expression, suggesting distinct underlying cellular mechanisms influenced by duration, time of exposure, and concentration.

Based on the quantification of adherent cells following exposure to different concentrations of ENNB1 (0.5–6 μM), a biphasic trend was observed: Initially, ENNB1 demonstrated a moderate yet significant increase in the number of adherent PAM viability after 6 h of incubation, particularly at the highest concentration (6 μM), resulting in a 39.5% increase compared to untreated cells. This effect became more evident at 24 h, with all tested concentrations (0.5–6 μM) significantly increasing crystal violet retention, indicating that ENNB1 may elicit early pro‐survival or cell‐protective responses.

This dose range likely reflects an optimal window for ENNB1‐induced activation of adaptive stress mechanisms, such as enhanced mitochondrial activity, antioxidant signaling, or moderate immune stimulation, possibly through mild oxidative stress (Gammelsrud et al. 2012; Prosperini et al. 2017). Such responses are indicative of an early adaptive or compensatory cellular response, characterized by transient activation of metabolic, stress, and immune‐related pathways at moderate concentrations and short exposure times, followed by functional impairment and cytotoxicity upon prolonged exposure (Mattson 2008). While this activation includes pro‐inflammatory cytokines whose sustained induction could be detrimental in vivo, in the in vitro context it indicates functional macrophage activation in response to xenobiotic stress (Ivanova et al. 2019). The slight reduction in adherent cell density at 6 μM compared to 3 μM at 24 h may represent the onset of stress saturation, suggesting a threshold beyond which protective responses begin to falter.

However, after 48 h, a concentration‐dependent reduction in the number of adherent cells was observed, with significant reduction starting at concentrations ≥ 1.5 μM. This temporal transition from enhanced adherence to cell loss suggests that ENNB1 initially engages protective cellular mechanisms, but long‐term exposure overwhelms these systems, leading to mitochondrial dysfunction, oxidative damage, and potentially apoptosis or necrosis (Cimbalo et al. 2021; Huang et al. 2019; Oliveira et al. 2020).

In support of these findings, the MTT assay provided complementary evidence of ENNB1‐induced modulation of metabolic activity over time. After 6 h of exposure, ENNB1 promoted a mild increase in mitochondrial activity, particularly at 1.5–6 μM, aligning with the early enhancement in cell adherence and suggesting transient stimulation of mitochondrial function. This short‐term increase may reflect a compensatory mitochondrial response or mild stress‐induced activation of cellular bioenergetics, as previously observed for other ENNs and structurally related ionophoric mycotoxins (Tonshin et al. 2010). At 24 h, this stimulatory effect became more pronounced, with a clear dose‐dependent elevation in metabolic activity, suggesting that ENNB1 may initially enhance cellular energy metabolism and stress‐adaptation pathways prior to the onset of cytotoxic effects (Fernández‐Blanco et al. 2016; Jonsson et al. 2016).

However, consistent with the decline in adherent cell numbers observed at longer exposure times, MTT results at 48 h revealed a marked and concentration‐dependent decrease in viability, with significant impairment beginning at 1.5 μM. This late reduction in metabolic activity further supports the shift toward cytotoxicity, in line with evidence that prolonged exposure to ENNs disrupts mitochondrial membrane potential, induces reactive oxygen species accumulation, and activates apoptotic pathways (Jonsson et al. 2016; Meca et al. 2011). Together, these findings corroborate the hypothesis that long‐term ENNB1 exposure exceeds cellular compensatory capacity, ultimately leading to mitochondrial dysfunction and loss of metabolic viability.

Gene expression analysis supports and expands upon the viability data, revealing molecular mechanisms that help explain the observed outcomes. At 24 h, a broad upregulation of immune‐related genes was observed, particularly at concentrations that promoted higher levels of adherent cells (1.5–3 μM). Pro‐inflammatory cytokines, such as IL‐1β, IL‐6, IL‐8, IL‐17A, and TNF‐α, were significantly upregulated, suggesting acute inflammatory activation in response to ENNB1. This may reflect an early “alarm” phase, where macrophages respond to a perceived threat by triggering innate immune responses (Gammelsrud et al. 2012). This activation is not necessarily detrimental; indeed, it may support the observed early cytoprotective responses by promoting cellular communication, tissue surveillance, and metabolic readiness (Huang et al. 2019).

Simultaneously, anti‐inflammatory cytokines IL‐10 and TGF‐β were also significantly upregulated, indicating a nuanced and self‐regulating immune response. The co‐expression of these antagonistic signals suggests an immunomodulatory role for ENNB1, where inflammatory activation is tightly controlled to prevent excessive damage (Gammelsrud et al. 2012; Oliveira et al. 2020).

Moreover, the induction of COX‐1 and COX‐2 enzymes at 24 h suggests increased prostaglandin synthesis, contributing to the inflammatory phenotype (Gammelsrud et al. 2012; Huang et al. 2019). GPX‐2, an antioxidant enzyme, was also significantly upregulated at 3 μM, likely reflecting a protective response against mild ENNB1‐induced oxidative stress (Oliveira et al. 2020; Prosperini et al. 2017). These patterns suggest that the 24‐h timepoint captures an integrated stress‐adaptive phase where ENNB1 stimulates both immune and redox‐regulatory networks.

In support of these findings, the transcriptional activation of key signaling mediators further highlights the involvement of major inflammatory pathways. STAT3 was markedly upregulated at 24 h, particularly at intermediate ENNB1 concentrations, consistent with its established role as a central regulator of cytokine‐driven macrophage activation and survival signaling (Yu et al. 2009; Villarino et al. 2017). STAT3 activation is commonly associated with IL‐6 and IL‐10 signaling and contributes to the coordination of inflammatory and compensatory anti‐inflammatory responses, reinforcing the concept of a tightly regulated activation state rather than uncontrolled inflammation.

Concomitantly, RELA p65 and NF‐κB were significantly induced at 24 h, confirming activation of the canonical NF‐κB pathway. NF‐κB is a master regulator of innate immunity and controls the transcription of multiple cytokines and enzymes identified as upregulated in this study, including IL‐1β, IL‐6, IL‐8, TNF‐α, and COX‐2 (Hayden and Ghosh 2014; Liu et al. 2017). The parallel upregulation of IκBα further supports this interpretation, as IκBα is a classical NF‐κB target gene that mediates negative feedback by restraining prolonged NF‐κB activation (Sun et al. 2013). Together, these results indicate that ENNB1 triggers a coordinated and self‐limiting inflammatory signaling cascade at early time points.

By contrast, at 48 h, gene expression patterns shifted substantially. Many cytokines and enzymes that were previously upregulated became downregulated or silenced, particularly at higher concentrations of the stimulus. IL‐1β, IL‐6, and TNF‐α, for example, showed reduced or inhibited expression, indicating that long‐term ENNB1 exposure disrupts immune signaling (Gammelsrud et al. 2012). At 24 h, IL‐6 expression at 6 μM was almost completely absent; this marked suppression is likely attributable, at least in part, to cytotoxic effects at this concentration, although impaired NF‐κB signaling or mitochondrial dysfunction cannot be excluded. At 48 h, a modest increase in expression was observed; however, given the very low absolute expression levels and the reduced cell viability at this time point, the biological relevance of this apparent rebound remains uncertain. Similar transient responses under conditions of cellular stress have been reported in other experimental models (Cimbalo et al. 2021; Ivanova et al. 2019), and this observation should therefore be interpreted with caution. Likewise, the diminished expression of COX‐1, COX‐2, and GPX‐2 at 48 h suggests that both inflammatory and antioxidant capacities are compromised under sustained stress (Huang et al. 2019; Oliveira et al. 2020).

At the signaling level, the marked downregulation of STAT3, RELA p65, and NF‐κB at 48 h indicates a collapse of transcriptional programs essential for macrophage activation and survival. In contrast, IκBα expression remained elevated, suggesting persistent repression of NF‐κB activity. Sustained IκBα accumulation has been associated with immune exhaustion, transcriptional silencing, and impaired responsiveness in macrophages exposed to chronic stressors (Sun et al. 2013; Lawrence 2009). This imbalance between inhibitory and activating signals likely contributes to the widespread suppression of immune gene expression observed at this later time point.

This widespread downregulation is consistent with the loss of adherent cells observed in the viability assays and may be attributed to energy depletion, transcriptional repression, or epigenetic silencing triggered by prolonged cellular distress. Because PAMs are terminally differentiated and nonproliferative under in vitro conditions, the reduced crystal violet staining observed at 48 h is unlikely to result from increased cell division leading to surface overcrowding. Rather, it is more consistent with a decline in viable adherent cells and reduced transcriptional activity. Nevertheless, future studies including proliferation and cell death markers will be required to confirm this interpretation. The loss of both pro‐ and anti‐inflammatory signaling implies a breakdown in immune homeostasis, possibly leading to immunosuppression or anergy.

Taken together, these findings suggest that ENNB1 interacts with macrophages through a dynamic and concentration/time‐dependent mechanism. Initially, ENNB1 may act as a mild stressor that activates immune surveillance, antioxidant defenses, and pro‐survival pathways, resulting in increased cell adherence. However, when exposure exceeds cellular coping capacities, either through higher doses or longer exposure, ENNB1 shifts from a modulator to a disruptor, impairing essential functions and triggering cell death pathways.

Furthermore, because ENNB1 appears to influence cholesterol biosynthesis (Coulet et al. 2024) through the inhibition of the acyl‐CoA:cholesterol acyltransferase (ATAC) enzyme (Sy‐Cordero et al. 2012), resulting in the accumulation of free cholesterol that influences the regulation of the inflammatory response (Bauer et al. 2023; Li et al. 2005), further investigations are needed to clarify the correct toxicity pathway.

This biphasic response likely involves mitochondrial perturbation and oxidative overload, ultimately resulting in transcriptional shutdown and metabolic failure (Dhalla et al. 2025; Guo et al. 2013). These features emphasize the importance of dose thresholds and temporal dynamics in evaluating the biological effects of ENNB1.

ENNB1 exerts a sophisticated and dualistic influence on PAMs. At intermediate concentrations and early time points, it promotes cell adhesion and survival‐associated responses, together with a balanced modulation of immune gene expression. However, with prolonged exposure or higher doses, these initially adaptive responses are lost, and cellular dysfunction emerges. This shift is likely driven by mechanisms involving oxidative stress, where excessive reactive oxygen species (ROS) accumulate and damage cellular structures, and immune suppression, characterized by the downregulation of key inflammatory and regulatory genes. Together, these processes compromise cellular homeostasis, reduce the cell's ability to respond to stimuli, and ultimately lead to cell death. These insights are crucial for assessing the potential risks of ENNB1 exposure in agricultural or biomedical contexts. Future studies should aim to dissect the precise molecular pathways, such as the involvement of specific receptors, transcription factors, or epigenetic modifiers, that govern this biphasic behavior.

## 5 Conclusion

This study provides novel insights into the immunomodulatory effects of ENNB1, an emerging mycotoxin, on PAMs, highlighting its biphasic and concentration‐/time‐dependent behavior. At low to intermediate concentrations and short‐term exposure times, ENNB1 enhanced cell retention and stimulated a coordinated transcriptional activation of both pro‐ and anti‐inflammatory genes, suggesting an early adaptive or compensatory response. Conversely, prolonged exposure or higher concentrations resulted in a marked reduction in cell adherence and a broad downregulation of immune and antioxidant gene expression, consistent with cytotoxicity, oxidative stress, and potential immunosuppressive effects. ENNB1‐induced changes in PAM viability and cell density were initially assessed using crystal violet staining, and independently corroborated by complementary MTT assays, which confirmed the concentration‐ and time‐dependent modulation of PAM metabolic activity and supported the interpretation of ENNB1‐induced cytotoxic and immunomodulatory effects. Therefore, while crystal violet staining alone should be interpreted with caution, the combined use of adherence‐based and metabolic assays provides a robust basis for the conclusions drawn in this study. Nonetheless, the porcine PAM model proved to be a valuable and physiologically relevant tool for exploring the immunotoxicological profile of ENNB1 in vitro. These findings emphasize the relevance of a species‐specific approach for risk assessment evaluation, particularly in the context of respiratory exposure. Further studies are warranted to delineate the underlying molecular pathways, including those involved in mitochondrial function, redox regulation, and immune signaling, to better understand the potential implications of ENNB1 exposure for animal and human health. Moreover taking together, these results also provide relevant insights for human health risk assessment. The biphasic immunomodulatory response observed in PAMs, initial immune activation followed by suppression and cytotoxicity, suggests that ENNB1 has the potential to disrupt immune homeostasis. This finding is of concern both in the context of dietary exposure through contaminated food and in scenarios of inhalation or dermal exposure, suggesting that immunotoxicity should be considered a critical endpoint in future ENNB1 risk assessment.

## Author Contributions

**Francesca Caloni**, **Sara Frazzini**, and **Luciana Rossi:** conceptualization. **Francesca Caloni** and **Sara Frazzini:** methodology. **Sara Frazzini:** software. **Luciana Rossi:** validation. **Sara Frazzini:** formal analysis. **Sara Frazzini:** investigation. **Francesca Caloni** and **Luciana Rossi:** resources. **Sara Frazzini:** data curation. **Sara Frazzini:** writing – original draft preparation. **Francesca Caloni**, **Paola Fossati**, **Giancarlo Ruffo**, and **Luciana Rossi:** writing – review and editing. **Sara Frazzini**, **Paola Fossati**, and **Luciana Rossi:** visualization. **Francesca Caloni**, **Giancarlo Ruffo**, and **Luciana Rossi:** supervision. **Luciana Rossi:** project administration. All authors have read and agreed to the published version of the manuscript.

## Conflicts of Interest

The authors declare no conflicts of interest.

## Supporting information


**Figure S1:** Representative phase‐contrast images of crystal violet‐stained porcine alveolar macrophages (PAMs) after exposure to increasing concentrations of Enniatin B1 (ENNB1). (a) 0 μM after 6 h of exposure, (b) 6 μM after 6 h of exposure, (c) 1.5 μM after 24 h of exposure, (d) 3 μM after 24 h of exposure, (e) 3 μM after 48 h of exposure, and (f) 6 μM after 48 h of exposure. Scale bar: 200 μm.

## Data Availability

The data that support the findings of this study are available from the corresponding author upon reasonable request.

## References

[jat70114-bib-0001] Balmelli, C. , N. Ruggli , K. McCullough , and A. Summerfield . 2005. “Fibrocytes Are Potent Stimulators of Anti‐Virus Cytotoxic T Cells.” Journal of Leukocyte Biology 77, no. 6: 923–933. 10.1189/jlb.1204701. 15767291

[jat70114-bib-0002] Bauer, R. , B. Brüne , and T. Schmid . 2023. “Cholesterol Metabolism in the Regulation of Inflammatory Responses.” Frontiers in Pharmacology 14: 1121819. 10.3389/fphar.2023.1121819. 36744258 PMC9895399

[jat70114-bib-0003] Behr, A.‐C. , C. K. Fæste , A. Azqueta , et al. 2025. “Hazard Characterization of the Mycotoxins Enniatins and Beauvericin to Identify Data Gaps and Improve Risk Assessment for Human Health.” Archives of Toxicology 99, no. 5: 1791–1841. 10.1007/s00204-025-03988-3. 40137953 PMC12085332

[jat70114-bib-0004] Caprarulo, V. , L. Turin , M. Hejna , et al. 2023. “Protective Effect of Phytogenic Plus Short and Medium‐Chain Fatty Acids‐Based Additives in Enterotoxigenic *Escherichia coli* Challenged Piglets.” Veterinary Research Communications 47, no. 1: 217–231. 10.1007/s11259-022-09945-0. 35616772 PMC9873745

[jat70114-bib-0005] Castell, A. , N. Arroyo‐Manzanares , N. Campillo , et al. 2025. “Reliable and Sensitive Analytical Platform to Assess Dietary Exposure of Pigs to Mycotoxins and Explore Potential Urinary Biomarkers.” Talanta 286: 127441. 10.1016/j.talanta.2024.127441. 39733520

[jat70114-bib-0006] Chen, D. , X. Liu , S. Xu , et al. 2019. “TNF‐α Induced by Porcine Reproductive and Respiratory Syndrome Virus Inhibits the Replication of Classical Swine Fever Virus C‐Strain.” Veterinary Microbiology 234: 25–33. 10.1016/j.vetmic.2019.05.007. 31213269

[jat70114-bib-0007] Chiminelli, I. , L. J. Spicer , E. R. S. Maylem , and F. Caloni . 2022. “Emerging Mycotoxins and Reproductive Effects in Animals: A Short Review.” Journal of Applied Toxicology 42, no. 12: 1901–1909. 10.1002/jat.4311. 35229323

[jat70114-bib-0008] Cimbalo, A. , M. Frangiamone , C. Juan , G. Font , M. Lozano , and L. Manyes . 2021. “Proteomics Evaluation of Enniatins Acute Toxicity in Rat Liver.” Food and Chemical Toxicology 151: 112130. 10.1016/j.fct.2021.112130. 33741480

[jat70114-bib-0009] Coulet, F. , M. Coton , C. Iperi , M. Belinger Podevin , E. Coton , and N. Hymery . 2024. “Cytotoxic Effects of Major and Emerging Mycotoxins on HepaRG Cells and Transcriptomic Response After Exposure of Spheroids to Enniatins B and B1.” Toxins 16, no. 1: 54. 10.3390/toxins16010054. 38251270 PMC10819306

[jat70114-bib-0010] Daniłowicz, E. , M. Akouchekian , C. Drögemüller , et al. 2008. “Molecular Characterization and SNP Development for the Porcine IL6 and IL10 Genes.” Animal Biotechnology 19, no. 3: 159–165. 10.1080/10495390802088621. 18607788

[jat70114-bib-0011] De Felice, B. , S. Mondellini , M. Bertazzo , M. Parolini , and F. Caloni . 2025. “Novel Miniaturized Exposure to Evaluate the Toxicity of Enniatin B1 on Daphnia Magna.” Environmental Toxicology and Pharmacology 117: 104748. 10.1016/j.etap.2025.104748. 40553954

[jat70114-bib-0012] De Felice, B. , L. J. Spicer , and F. Caloni . 2023. “Enniatin B1: Emerging Mycotoxin and Emerging Issues.” Toxins 15, no. 6: 383. 10.3390/toxins15060383. 37368684 PMC10303499

[jat70114-bib-0013] de Martin, R. , H. Holzmüller , E. Hofer , and F. H. Bach . 1995. “Intron‐Exon Structure of the Porcine I Kappa B Alpha‐Encoding Gene.” Gene 152, no. 2: 253–255. 10.1016/0378-1119(94)00726-9. 7835710

[jat70114-bib-0014] Dell'Anno, M. , S. Frazzini , S. Reggi , et al. 2024. “Evaluation of Dietary Supplementation of *Ascophyllum nodosum* and *Lithothamnium calcareum* as Functional Algae in F4+ *Escherichia coli* Challenged Piglets.” Frontiers in Veterinary Science 11: 1430347. 10.3389/fvets.2024.1430347. 39309030 PMC11412951

[jat70114-bib-0015] Dhalla, N. S. , P. Ostadal , and P. S. Tappia . 2025. “Involvement of Oxidative Stress in Mitochondrial Abnormalities During the Development of Heart Disease.” Biomedicine 13, no. 6: 1338. 10.3390/biomedicines13061338. PMC12189734 40564057

[jat70114-bib-0016] Dornetshuber, R. , P. Heffeter , M. Sulyok , et al. 2009. “Interactions Between ABC‐Transport Proteins and the Secondary *Fusarium* Metabolites Enniatin and Beauvericin.” Molecular Nutrition & Food Research 53, no. 7: 904–920. 10.1002/mnfr.200800384. 19517454

[jat70114-bib-0017] European Commission . 2010. Directive 2010/63/EU of the European Parliament and of the Council of 22 September 2010 on the Protection of Animals Used for Scientific Purposes Text With EEA relevance. Official Journal of the European Union.

[jat70114-bib-0018] Fairbairn, L. , R. Kapetanovic , D. P. Sester , and D. A. Hume . 2011. “The Mononuclear Phagocyte System of the Pig as a Model for Understanding Human Innate Immunity and Disease.” Journal of Leukocyte Biology 89, no. 6: 855–871. 10.1189/jlb.1110607. 21233410

[jat70114-bib-0072] Fernández‐Blanco, C. , G. Font , and M.‐J. Ruiz . 2016. “Interaction Effects of Enniatin B, Deoxinivalenol and Alternariol in Caco‐2 Cells.” Toxicology Letters 241: 38–48. 10.1016/j.toxlet.2015.11.005. 26581633

[jat70114-bib-0019] Fraeyman, S. , S. Croubels , M. Devreese , and G. Antonissen . 2017. “Emerging *Fusarium* and *Alternaria* Mycotoxins: Occurrence, Toxicity and Toxicokinetics.” Toxins 9, no. 7: 228. 10.3390/toxins9070228. 28718805 PMC5535175

[jat70114-bib-0020] Frazzini, S. , L. Turin , G. Vanosi , L. Rossi , and M. Hejna . 2025. “Seaweed‐Derived Mixed Extracts Exhibit Immunomodulatory Properties on Porcine Alveolar Macrophages.” Veterinary Journal 312: 106358. 10.1016/j.tvjl.2025.106358. 40246016

[jat70114-bib-0021] Gammelsrud, A. , A. Solhaug , B. Dendelé , et al. 2012. “Enniatin B‐Induced Cell Death and Inflammatory Responses in RAW 267.4 Murine Macrophages.” Toxicology and Applied Pharmacology 261, no. 1: 74–87. 10.1016/j.taap.2012.03.014. 22483798

[jat70114-bib-0022] Giannioti, Z. , B. Albero , M. D. Hernando , L. Bontempo , and R. A. Pérez . 2023. “Determination of Regulated and Emerging Mycotoxins in Organic and Conventional Gluten‐Free Flours by LC‐MS/MS.” Toxins 15, no. 2: 155. 10.3390/toxins15020155. 36828469 PMC9966797

[jat70114-bib-0023] Guo, C. , L. Sun , X. Chen , and D. Zhang . 2013. “Oxidative Stress, Mitochondrial Damage and Neurodegenerative Diseases.” Neural Regeneration Research 8, no. 21: 2003–2014. 10.3969/j.issn.1673-5374.2013.21.009. 25206509 PMC4145906

[jat70114-bib-0024] Hayden, M. S. , and S. Ghosh . 2014. “Regulation of NF‐κB by TNF Family Cytokines.” Immunological Reviews 252, no. 1: 25–45. 10.1016/j.smim.2014.05.004.

[jat70114-bib-0025] Huang, C. , F. Wang , and W. Chan . 2019. “Enniatin B1 Exerts Embryotoxic Effects on Mouse Blastocysts and Induces Oxidative Stress and Immunotoxicity During Embryo Development.” Environmental Toxicology 34, no. 1: 48–59. 10.1002/tox.22656. 30259633

[jat70114-bib-0027] Ivanova, L. , I. G. Denisov , Y. V. Grinkova , S. G. Sligar , and C. K. Fæste . 2019. “Biotransformation of the Mycotoxin Enniatin B1 by CYP P450 3A4 and Potential for Drug‐Drug Interactions.” Metabolites 9, no. 8: 158. 10.3390/metabo9080158. 31357617 PMC6724072

[jat70114-bib-0028] Ivanova, L. , S. Uhlig , M. Devreese , S. Croubels , and C. K. Fæste . 2017. “Biotransformation of the Mycotoxin Enniatin B1 in Pigs: A Comparative In Vitro and In Vivo Approach.” Food and Chemical Toxicology 105: 506–517. 10.1016/j.fct.2017.04.041. 28472676

[jat70114-bib-0026] Ivanova, L. , S. Uhlig , G. Eriksen , and L. Johannessen . 2010. “Enniatin B1 Is a Substrate of Intestinal P‐Glycoprotein, Multidrug Resistance‐Associated Protein 2 and Breast Cancer Resistance Protein.” World Mycotoxin Journal 3, no. 3: 271–281. 10.3920/WMJ2010.1225.

[jat70114-bib-0029] Ji, X. , Y. Zhou , Y. Xiao , et al. 2024. “A Tiered Approach of Hazard‐Prioritization and Risk‐Ranking for Chemical Hazards in Food Commodities: Application for Selected Mycotoxins.” Food Research International 178: 113946. 10.1016/j.foodres.2024.113946. 38309871

[jat70114-bib-0073] Jonsson, M. , M. Jestoi , M. Anthoni , et al. 2016. “Fusarium Mycotoxin Enniatin B: Cytotoxic Effects and Changes in Gene Expression Profile.” Toxicology in Vitro 34: 309–320. 10.1016/j.tiv.2016.04.017. 27163883

[jat70114-bib-0030] Khatua, S. , J. Simal‐Gandara , and K. Acharya . 2022. “Understanding Immune‐Modulatory Efficacy In Vitro.” Chemico‐Biological Interactions 352: 109776. 10.1016/j.cbi.2021.109776. 34906553 PMC8665649

[jat70114-bib-0031] Kijas, J. M. H. , and L. Andersson . 2001. “A Phylogenetic Study of the Origin of the Domestic Pig Estimated From the Near‐Complete mtDNA Genome.” Journal of Molecular Evolution 52, no. 3: 302–308. 10.1007/s002390010158. 11428467

[jat70114-bib-0032] Lawrence, T. 2009. “The Nuclear Factor NF‐κB Pathway in Inflammation.” Cold Spring Harbor Perspectives in Biology 1, no. 6: a001651. 10.1101/cshperspect.a001651. 20457564 PMC2882124

[jat70114-bib-0033] Li, B. , J. Fang , Z. Zuo , et al. 2018. “Activation of Porcine Alveolar Macrophages by *Actinobacillus pleuropneumoniae* Lipopolysaccharide via the Toll‐Like Receptor 4/NF‐κB‐Mediated Pathway.” Infection and Immunity 86, no. 3: e00617–e00642. 10.1128/IAI.00642-17. PMC5820947 29229731

[jat70114-bib-0034] Li, Y. , R. F. Schwabe , T. DeVries‐Seimon , et al. 2005. “Free Cholesterol‐Loaded Macrophages Are an Abundant Source of Tumor Necrosis Factor‐α and Interleukin‐6.” Journal of Biological Chemistry 280, no. 23: 21763–21772. 10.1074/jbc.M501759200. 15826936

[jat70114-bib-0035] Liu, T. , L. Zhang , D. Joo , and S. C. Sun . 2017. “NF‐κB Signaling in Inflammation.” Signal Transduction and Targeted Therapy 2: 17023. 10.1038/sigtrans.2017.23. 29158945 PMC5661633

[jat70114-bib-0036] Luo, Y. , X. Zhang , Z. Zhu , N. Jiao , K. Qiu , and J. Yin . 2018. “Surplus Dietary Isoleucine Intake Enhanced Monounsaturated Fatty Acid Synthesis and Fat Accumulation in Skeletal Muscle of Finishing Pigs.” Journal of Animal Science and Biotechnology 9, no. 1: 88. 10.1186/s40104-018-0306-5. 30598820 PMC6302484

[jat70114-bib-0037] Mattson, M. P. 2008. “Hormesis Defined.” Ageing Research Reviews 7, no. 1: 1–7. 10.1016/j.arr.2007.08.007. 18162444 PMC2248601

[jat70114-bib-0038] Meca, G. , G. Font , and M. J. Ruiz . 2011. “Comparative Cytotoxicity Study of Enniatins A, A1, A2, B, B1, B4 and J3 on Caco‐2 Cells, Hep‐G2 and HT‐29.” Food and Chemical Toxicology 49, no. 9: 2464–2469. 10.1016/j.fct.2011.05.020. 21640785

[jat70114-bib-0039] Meurens, F. , A. Summerfield , H. Nauwynck , L. Saif , and V. Gerdts . 2012. “The Pig: A Model for Human Infectious Diseases.” Trends in Microbiology 20, no. 1: 50–57. 10.1016/j.tim.2011.11.002. 22153753 PMC7173122

[jat70114-bib-0040] Oh, D. , W. De Spiegelaere , and H. J. Nauwynck . 2023. “Selection and Validation of Reference Genes for RT‐qPCR Normalization of Porcine Alveolar Macrophages (PAMs) for PRRSV Studies.” Scientific Reports 13, no. 1: 8840. 10.1038/s41598-023-35873-3. 37258711 PMC10232543

[jat70114-bib-0041] Oliveira, C. A. F. , L. Ivanova , A. Solhaug , and C. K. Fæste . 2020. “Enniatin B1‐Induced Lysosomal Membrane Permeabilization in Mouse Embryonic Fibroblasts.” Mycotoxin Research 36, no. 1: 23–30. 10.1007/s12550-019-00366-8. 31264166

[jat70114-bib-0042] Pabst, R. 2020. “The Pig as a Model for Immunology Research.” Cell and Tissue Research 380, no. 2: 287–304. 10.1007/s00441-020-03206-9. 32356014 PMC7223737

[jat70114-bib-0043] Pérez‐Fuentes, N. , R. Alvariño , A. Alfonso , et al. 2021. “Single and Combined Effects of Regulated and Emerging Mycotoxins on Viability and Mitochondrial Function of SH‐SY5Y Cells.” Food and Chemical Toxicology 154: 112308. 10.1016/j.fct.2021.112308. 34062223

[jat70114-bib-0044] Pérez‐Fuentes, N. , R. Alvariño , A. Alfonso , et al. 2022. “Enniatins A1 and B1 Alter Calcium Homeostasis of Neuronal Cells Leading to Apoptotic Death.” Food and Chemical Toxicology 168: 113361. 10.1016/j.fct.2022.113361. 35970269

[jat70114-bib-0045] Pérez‐Fuentes, N. , R. Alvariño , A. Alfonso , J. González‐Jartín , M. R. Vieytes , and L. M. Botana . 2024a. “Enniatins A1 and B1 Modulate Calcium Flux Through Alternative Pathways Beyond Mitochondria.” Journal of Agricultural and Food Chemistry 72, no. 26: 14975–14983. 10.1021/acs.jafc.4c04242. 38898562 PMC11229004

[jat70114-bib-0046] Pérez‐Fuentes, N. , R. Alvariño , A. Alfonso , J. González‐Jartín , M. R. Vieytes , and L. M. Botana . 2024b. “In Vitro Assessment of Emerging Mycotoxins Co‐Occurring in Cheese: A Potential Health Hazard.” Archives of Toxicology 98, no. 12: 4173–4186. 10.1007/s00204-024-03872-6. 39322822

[jat70114-bib-0047] Pérez‐Fuentes, N. , R. Alvariño , A. Alfonso , J. González‐Jartín , M. R. Vieytes , and L. M. Botana . 2024c. “The Mode of Action of Enniatins A and B is Mediated by Interaction With SOC Reservoirs (A) and Mitochondrial Permeability Transition Pore (B).” Exposure and Health 16, no. 4: 1115–1126. 10.1007/s12403-023-00613-5.

[jat70114-bib-0049] Prosperini, A. , H. Berrada , M. J. Ruiz , et al. 2017. “A Review of the Mycotoxin Enniatin B.” Frontiers in Public Health 5: 304. 10.3389/fpubh.2017.00304. 29201864 PMC5697211

[jat70114-bib-0048] Prosperini, A. , G. Font , and M. J. Ruiz . 2014. “Interaction Effects of Fusarium Enniatins (A, A1, B and B1) Combinations on In Vitro Cytotoxicity of Caco‐2 Cells.” Toxicology In Vitro 28, no. 1: 88–94. 10.1016/j.tiv.2013.06.021. 23850737

[jat70114-bib-0050] Rodríguez‐Cañás, I. , J. M. González‐Jartín , R. Alvariño , A. Alfonso , M. R. Vieytes , and L. M. Botana . 2023. “Detection of Mycotoxins in Cheese Using an Optimized Analytical Method Based on a QuEChERS Extraction and UHPLC‐MS/MS Quantification.” Food Chemistry 408: 135182. 10.1016/j.foodchem.2022.135182. 36535186

[jat70114-bib-0051] Ryan, M. T. , C. J. O'Shea , C. B. Collins , J. V. O'Doherty , and T. Sweeney . 2012. “Effects of Dietary Supplementation With *Laminaria hyperborea*, *Laminaria digitata*, and *Saccharomyces cerevisiae* on the IL‐17 Pathway in the Porcine Colon.” Journal of Animal Science 90, no. suppl_4: 263–265. 10.2527/jas.53802. 23365350

[jat70114-bib-0052] Santini, A. , G. Meca , S. Uhlig , and A. Ritieni . 2012. “Fusaproliferin, Beauvericin and Enniatins: Occurrence in Food – A Review.” World Mycotoxin Journal 5, no. 1: 71–81. 10.3920/WMJ2011.1331.

[jat70114-bib-0053] Shen, H. , Y. Cai , K. Zhu , D. Wang , R. Yu , and X. Chen . 2024. “Enniatin B1 Induces Damage to Leydig Cells via Inhibition of the Nrf2/HO‐1 and JAK/STAT3 Signaling Pathways.” Ecotoxicology and Environmental Safety 273: 116116. 10.1016/j.ecoenv.2024.116116. 38387140

[jat70114-bib-0054] Smolinska, N. , K. Szeszko , K. Dobrzyn , et al. 2019. “Transcriptomic Analysis of Porcine Endometrium During Implantation After In Vitro Stimulation by Adiponectin.” International Journal of Molecular Sciences 20, no. 6: 1335. 10.3390/ijms20061335. 30884816 PMC6470965

[jat70114-bib-0055] Sun, S. C. , J. H. Chang , and J. Jin . 2013. “Regulation of Nuclear Factor‐κB in Autoimmunity.” Trends in Immunology 34, no. 6: 282–289. 10.1016/j.it.2013.01.004. 23434408 PMC3664242

[jat70114-bib-0056] Sy‐Cordero, A. A. , C. J. Pearce , and N. H. Oberlies . 2012. “Revisiting the Enniatins: A Review of Their Isolation, Biosynthesis, Structure Determination and Biological Activities.” Journal of Antibiotics 65, no. 11: 541–549. 10.1038/ja.2012.71. 22990381 PMC3573854

[jat70114-bib-0071] Tonshin, A. A. , V. V. Teplova , M. A. Andersson , and M. S. Salkinoja‐Salonen . 2010. “The Fusarium Mycotoxins Enniatins and Beauvericin Cause Mitochondrial Dysfunction by Affecting the Mitochondrial Volume Regulation, Oxidative Phosphorylation and Ion Homeostasis.” Toxicology 276, no. 1: 49–57. 10.1016/j.tox.2010.07.001. 20621153

[jat70114-bib-0057] Villarino, A. V. , Y. Kanno , J. R. Ferdinand , and J. J. O'Shea . 2017. “Mechanisms of Jak/STAT Signaling in Immunity and Disease.” Journal of Immunology 198, no. 5: 1803–1815. 10.4049/jimmunol.1401867. PMC4524500 25527793

[jat70114-bib-0058] Yang, L. , R. Wang , Z. Ma , et al. 2017. “Porcine Reproductive and Respiratory Syndrome Virus Antagonizes JAK/STAT3 Signaling via nsp5, Which Induces STAT3 Degradation.” Journal of Virology 91, no. 3: e02087–e02016. 10.1128/JVI.02087-16. 27881658 PMC5244345

[jat70114-bib-0059] Yu, H. , D. Pardoll , and R. Jove . 2009. “STATs in Cancer Inflammation and Immunity: A Leading Role for STAT3.” Nature Reviews Cancer 9, no. 11: 798–809. 10.1038/nrc2734. 19851315 PMC4856025

[jat70114-bib-0060] Zeitz, J. O. , S. Kaltenböck , E. Most , and K. Eder . 2017. “Antioxidant Status and Expression of Inflammatory Genes in Gut and Liver of Piglets Fed Different Dietary Methionine Concentrations.” Journal of Animal Physiology and Animal Nutrition 101, no. 6: 1166–1174. 10.1111/jpn.12633. 28066942

