# Supplementary material for: Immunomodulatory and Cytotoxic Properties of Enniatin B1 in Porcine Alveolar Macrophages
Source: J Appl Toxicol. 2026 Feb 19;46(9):3220–31. doi: 10.1002/jat.70114 (PMC13432692; doi:10.1002/jat.70114)

**Supplementary Figure 1:** Representative phase-contrast images of crystal violet-stained porcine alveolar macrophages (PAMs) after exposure to increasing concentrations of Enniatin B1 (ENNB1). (a) 0 µM after 6 hours of exposure, (b) 6 µM after 6 hours of exposure, (c) 1.5 µM after 24 hours of exposure, (d) 3 µM after 24 hours of exposure, (e) 3 µM after 48 hours of exposure, (f) 6 µM after 48 hours of exposure. Scale bar: 200 µm.


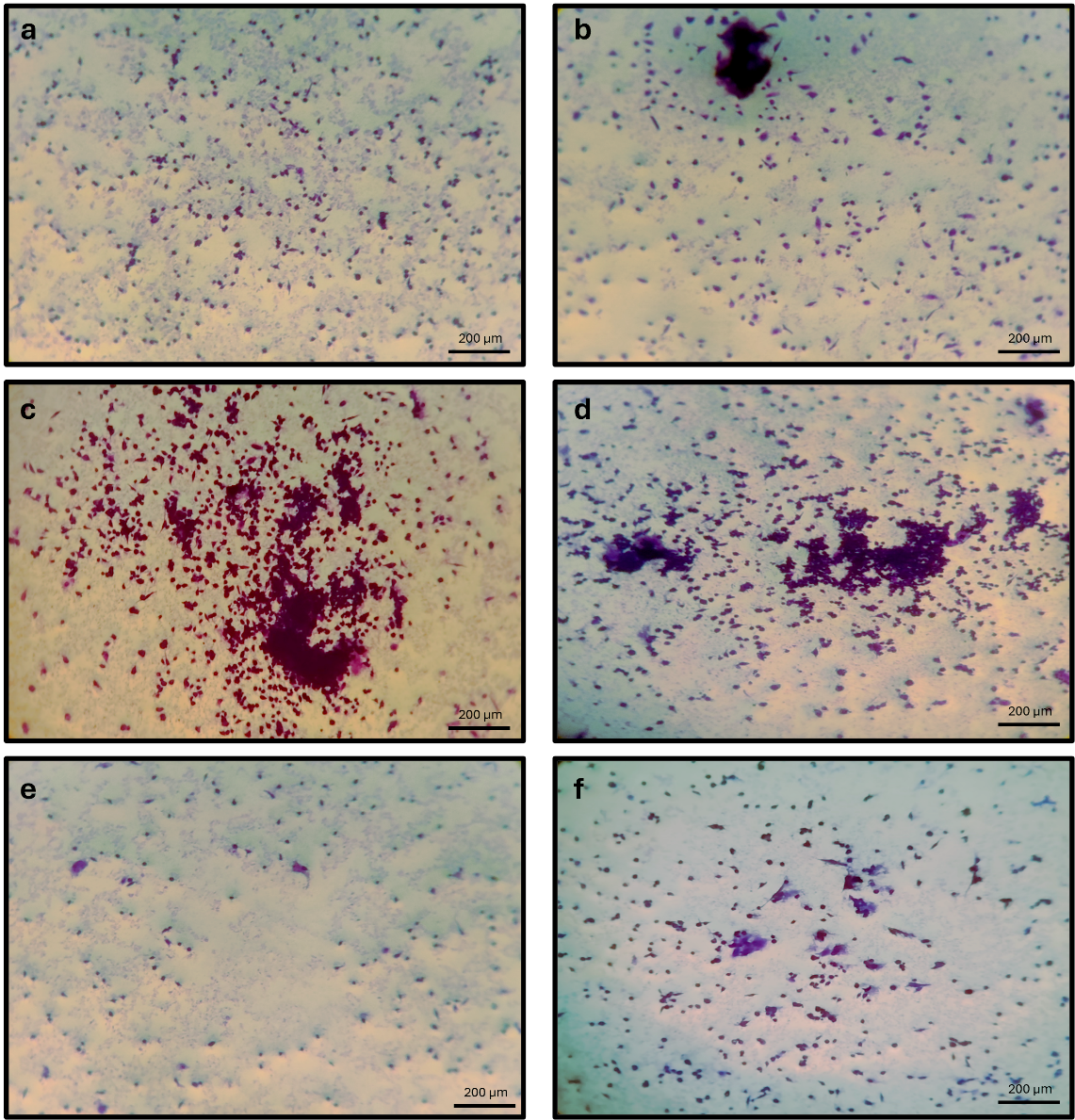

Supplement: Supplementary file 1 — Figure S1: Representative phase‐contrast images of crystal violet‐stained porcine alveolar macrophages (PAMs) after exposure to increasing concentrations of Enniatin B1 (ENNB1). (a) 0 μM after 6 h of exposure, (b) 6 μM after 6 h of exposure, (c) 1.5 μM after 24 h of exposure, (d) 3 μM after 24 h of exposure, (e) 3 μM after 48 h of exposure, and (f) 6 μM after 48 h of exposure. Scale bar: 200 μm. [file JAT-46-3220-s001.docx]
